# Differential Gut Microbiota and Fecal Metabolites Related With the Clinical Subtypes of Myasthenia Gravis

**DOI:** 10.3389/fmicb.2020.564579

**Published:** 2020-09-08

**Authors:** Xunmin Tan, Yu Huang, Tingjia Chai, Xiaoli Zhao, Yifan Li, Jing Wu, Hanping Zhang, Jiajia Duan, Weiwei Liang, Bangmin Yin, Ke Cheng, Gang Yu, Peng Zheng, Peng Xie

**Affiliations:** ^1^Department of Neurology, The First Affiliated Hospital of Chongqing Medical University, Chongqing, China; ^2^NHC Key Laboratory of Diagnosis and Treatment on Brain Functional Diseases, Chongqing Medical University, Chongqing, China; ^3^Chongqing Key Laboratory of Neurobiology, Chongqing, China; ^4^The M.O.E. Key Laboratory of Laboratory Medical Diagnostics, The College of Laboratory Medicine, Chongqing Medical University, Chongqing, China; ^5^Department of Neurology, Yongchuan Hospital, Chongqing Medical University, Chongqing, China

**Keywords:** myasthenia gravis, clinical subtypes, gut microbiota, metabolome, biomarker panels

## Abstract

Myasthenia gravis (MG) is a devastating acquired autoimmune disease. Previous studies have observed that disturbances of gut microbiome may attribute to the development of MG through fecal metabolomic signatures in humans. However, whether there were differential gut microbial and fecal metabolomic phenotypes in different subtypes of MG remains unclear. Here, our objective was to explore whether the microbial and metabolic signatures of ocular (OMG) and generalized myasthenia gravis (GMG) were different, and further identify the shared and distinct markers for patients with OMG and GMG. In this study, 16S ribosomal RNA (rRNA) gene sequencing and gas chromatography-mass spectrometry (GC/MS) were performed to capture the microbial and metabolic signatures of OMG and GMG, respectively. Random forest (RF) classifiers was used to identify the discriminative markers for OMG and GMG. Compared with healthy control (HC) group, GMG group, but not OMG group, showed a significant decrease in α-phylogenetic diversity. Both OMG and GMG groups, however, displayed significant gut microbial and metabolic disorders. Totally, we identified 20 OTUs and 9 metabolites specific to OMG group, and 23 OTUs and 7 metabolites specific to GMG group. Moreover, combinatorial biomarkers containing 15 discriminative OTUs and 2 differential metabolites were capable of discriminating OMG and GMG from each other, as well as from HCs, with AUC values ranging from 0.934 to 0.990. In conclusion, different subtypes of MG harbored differential gut microbiota, which generated discriminative fecal metabolism.

## Introduction

Myasthenia gravis (MG) is an autoimmune disease caused by autoantibodies that target the neuromuscular junction (NMJ), leading to partial or systemic muscle weakness and abnormal fatigability (Gilhus et al., 2016). Based on the location of the affected muscles, patients with MG are classified as ocular (OMG) or generalized MG (GMG) (Qiu et al., 2018). Ocular myasthenia gravis (OMG) is distinguished by fatigable weakness of the extraocular muscles, eyelids, or both, leading to fatigable ptosis and diplopia (Vaphiades et al., 2012). Almost 30–80% of patients with OMG would convert to generalized myasthenia gravis (GMG) within 2 years (Wong et al., 2016). These subjects not only suffer from ptosis and diplopia but also from limb weakness, bulbar symptoms, or even respiratory failure (Apinyawasisuk et al., 2019). Therefore, early recognition and treatment are of great importance for patients’ quality of life.

Although standard diagnostic tests for MG have good specificity, they are generally hindered by low sensitivity (thus high negative predictive value), particularly in cases of isolated OMG (Benatar, 2006). For example, acetylcholine receptor and MUSK antibodies have low sensitivity (approximately 0.38–0.71 for OMG, compared to 0.87–0.98 for GMG), although recent data suggest the sensitivity may be slightly higher than previously reported (Peeler et al., 2015). Repetitive nerve stimulation also has poor sensitivity (0.11–0.39 for OMG,0.53–0.98 for GMG). Single-fiber EMG has higher sensitivity (0.62–0.83 for OMG, 0.75–0.98 for GMG), but is a technically challenging procedure with limited availability (Benatar, 2006). Meanwhile, in the absence of reliable confirmatory tests, the diagnosis of OMG is often delayed. Furthermore, the physicians must often consider whether to pursue empiric treatment with agents including pyridostigmine, corticosteroids, and sometimes other immuno-suppressant drugs (Dalakas, 2019); and the toxicities associated with these medications are not trivial. The improved diagnostic sensitivity can help patients with MG to choose the right treatment. Therefore, a new test that retains high specificity while offering improved sensitivity would be a welcome addition to the diagnostic evaluation available for these patients (Sashank Prasad, 2016).

The gastrointestinal (GI) tract is an intricate ecosystem harboring a large number of symbiotic microorganisms, of which genetic information is 50 to 100 times more than that of human beings (Whitman et al., 1998). Therefore, the gut microbiota is considered as the largest and most direct external environment for human, which plays an indispensable role in maintaining human health (Rooks and Garrett, 2016). In recent years, mounting evidences have shown that gut microbiota plays a crucial role in the onset of central [e.g., autism spectrum disorder (Hsiao et al., 2013; Sharon et al., 2019)] and peripheral [e.g., irritable bowel syndrome (Duerkop et al., 2018)] diseases. Meanwhile, previous studies have found that gut microbial disorders may attribute to the onset of MG patients through fecal metabolism (Qiu et al., 2018; Zheng et al., 2019a). For instance, previous studies have observed that patients with MG showed a decrease in α-phylogenetic diversity, and significantly altered gut microbiome and fecal metabolome. Furthermore, germ-free (GF) mice colonized with MG microbiota (MMb) showed substantially impaired locomotion ability relative to the mice colonized with healthy microbiota (HMb). And this effect could be effectively reversed by co-housing procedures (Zheng et al., 2019a). However, until now, whether there were significant differences of gut microbial and metabolomic phenotypes in different subtypes of MG patients (OMG and GMG) remains largely unknown.

To address this issue, the gut microbiome and fecal metabolome among OMG, GMG and HCs were compared. 16S ribosomal RNA (rRNA) gene sequencing is beneficial to describe the microbial composition (Yarza et al., 2014), but cannot explain the functional changes. Furthermore, Gas chromatography-mass spectrometry (GC–MS)-based metabolomics were performed to unravel the overall metabolite profiles of fecal samples. Thus, these two complemental methods were used to identify the shared and distinct markers for patients with OMG and GMG. In addition, we ought to determine the discriminative performance of these microbial and metabolic markers in different subtypes of MG.

## Materials and Methods

### Subject Recruitment and Sample Collection

All the data were derived from our previous investigation (Zheng et al., 2019a). Briefly, ptients with MG were diagnosed and classified based on previous literature (Cortes-Vicente et al., 2016). The quantitative myasthenia gravis (QMG) test was used to quantify the severity of MG (scores range from 0 to 39) (Wolfe et al., 2016). Moreover, MG subjects were classified as one of two subtypes including OMG cohort (class I, *n* = 31) and GMG cohort (class IIa and IIb,*n* = 39) based on modifications of Osserman’s (Jaretzki et al., 2000). At last, 31 OMG patients [age, 41.0 (27.0–67.0); sex, male: female, 18:13; BMI, 22.2 (19.5–25.2)] and 39 GMG patients [age, 45.0 (33.0–56.0); sex, male: female, 13:26; BMI, 22.9 (19.6–24.5)] were recruited. Similarly, 74 HCs [age, 41.5 (26.0–52.3); sex, male: female, 30:44] were recruited from the medical examination center of the First Affiliated Hospital at Chongqing Medical University (Table 1). Participants with hepatic and/or renal diseases, gastrointestinal tract disorders, tumor, metabolic diseases, psychiatric disorders or any other disease that could affect the results of the study were excluded. None of the individuals took any antibiotics, probiotics, or prebiotics within 1 month prior to sampling. Fresh midstream stool samples from the recruited subjects were collected with a sterile cup and then quickly transferred to the sterile tubes. All collected samples were preserved at 4°C during transportation. Then, we stored all the fecal samples at -80°C till subsequent processing. The study protocol was reviewed and approved by the ECCMU. All participants signed a written informed consent before any procedures were performed.

**TABLE 1:** Detailed clinical characteristics of the subjects.

**Characteristics**	**OMG**	**GMG**	**HC**	***P*-value^*a*^**
Sample size	31	39	74	–
Female, *n* (%)	13 (41.9%)	26 (66.7%)	44 (59.5%)	0.104
Age, median (IQR)^*b*^	41.0 (27.0–67.0)	45.0 (33.0–56.0)	41.5 (26.0–52.3)	0.166
BMI, median (IQR)	22.2 (19.5–25.2)	22.9 (19.6–24.5)	–	0.615
Duration of disease, median (IQR)	3.0 (1.5–10.0)	4.0 (1.0–6.0)	–	0.631
Thymoma/Thymic hyperplasia, *n* (%)	10 (32.2%)	13 (33.3%)	–	0.924
Thymectomy, *n* (%)	6 (19.4%)	10 (25.6%)	–	0.534
AChR antibody test, *n* (%)	9 (29.0%)	14 (35.9%)	–	0.544
Anti-AChR antibody (+), *n* (%)	6 (19.4%)	9 (23.1%)	–	0.706
Immunosuppressive treatment, *n* (%)	12 (38.7%)	19 (48.7%)	–	0.402
QMG, median (IQR)^*c*^	2 (1.0–3.0)	5 (2.0–8.0)	–	0.002**

OMG, the ocular myasthenia gravis; GMG, the generalized myasthenia gravis; HC, healthy control. ^*a*^Chi-square analyses for categorical variables (sex); non-parametric factorial Wilcoxon rank-sum/Kruskal–Wallis test for continuous variables (age). ^*b*^Values are expressed as median with interquartile range (IQR). ^*c*^Quantitative Myasthenia Gravis (QMG) test, QMG scores range from 0 to 39. *p < 0.05, **p < 0.01, and ***p < 0.001.

### DNA Extraction, PCR Amplification, and Illumina MiSeq Sequencing

Total microbial DNA was extracted from stool samples by means of the QIAamp^®^DNA Stool Mini Kit (Qiagen, Hilden, Germany) according to the manufacturer’s protocol. The V3-V4 regions of the bacterial 16S rRNA gene were amplified by PCR using primers 338F and 806R (Zheng et al., 2019b) which contained an eight-base sequence unique to each sample. PCR reactions were carried out in triplicate 20 μl mixtures. PCR products were extracted from 2% agarose gels, purified using the AxyPrep DNA Gel Extraction Kit (Axygen Biosciences, Union City, CA, United States) and quantified with QuantiFluor^TM^-ST (Promega, United States). The Illumina MiSeq sequencing protocol was according to our previous published literature (Zheng et al., 2019a). Briefly, paired-end sequenced (2 × 250) on an Illumina MiSeq platform according to the standard manufacturer’s protocols.

### 16S rRNA Gene Sequence Analysis

Raw FASTQ files were demultiplexed, and quality-filtered using QIIME (version 1.17^1^). The 250 bp reads were truncated at any site of more than three sequential bases receiving an average quality score < 20. Reads shorter than 50 bp containing ambiguous base calls or barcode/primer errors were removed. Chimeric sequences were checked by UCHIME^2^ and removed from subsequent analyses. Operational taxonomic units (OTUs) clustering was carried out at a 97% (Gevers et al., 2005; Grice et al., 2008) similarity threshold using usearch (version 7.0^3^). α-diversity was measured by microbial community richness (Chao, Ace) (Minamoto et al., 2015) and diversity (Shannon, Invsimpson) (Zheng et al., 2019b). Beta diversity were assessed using unweighted UniFrac algorithms and visualized by non-metric multidimensional scaling (NMDS) analysis (Yang et al., 2019). The key different OTUs responsible for discrimination among the three groups were identified using linear discriminant analysis (LDA) effect size (LEfSe) analysis (Riquelme et al., 2019). An implementation of LEfSe including a convenient graphical interface incorporated in the Galaxy framework (Blankenberg et al., 2010; Goecks et al., 2010) is provided online at LEfSe^4^ (Segata et al., 2011). We modified the default calculation by controlling the multiple testing using Benjamini–Hochberg (BH) false discovery rate (FDR) correction procedure. LEfSe analysis was conducted under the following conditions: *p* < 0.05, FDR < 0.1 and LDA > 2.5 (Erawijantari et al., 2020; Zheng et al., 2020).

### Fecal Metabolome Analysis

Fecal metabolomic analysis was performed using gas chromatography-mass spectrometry (GC/MS; Agilent 7890A/5975C). The acquired MS data from GC/MS were demultiplexed, and quality-filtered by ChromaTOF software (v 4.34, LECO, St. Joseph, MI, United States). Any known pseudo positive peaks, such as peaks caused by noise, column bleed and BSTFA derivatization procedure, were removed from the data set, and the peaks from the same metabolite were combined. The resulting data were imported into a SIMCA (version 14.0, Umetrics, Umeå, Sweden) to perform orthogonal partial least-squares discriminant analysis (OPLS-DA) (Chen et al., 2018). R2X, R2Y were used to assess the goodness-of-fit, and Q2 was used to assess the predictability of the model. In addition, using Benjamin Hochberg method (BH method), FDR values for multiple testing of differential fecal metabolites between OMG or GMG and control groups were calculated. By analysis of OPLS-DA loadings, the differential metabolites responsible for discriminating between the two groups were identified with *p* < 0.05, FDR < 0.1 and variable importance plot values (VIP) > 1.0 (Liu et al., 2016). Kyoto Encyclopedia of Genes and Genomes (KEGG) database was used to explore the molecular pathways and biological functions of the identified differential metabolites (Chou et al., 2009).

To identify combinatorial biomarkers across the OMG, GMG, and HC groups, the different OTUs and metabolites among the three groups were analyzed using LEfse and OPLS-DA analysis, respectively. Further, all discriminative OTUs and metabolites were input for the random forest classifier (Python’s scikit-learn package) to predict the discrimination OMG and GMG (from each other and from HC). In each case, 1000 trees were considered (other scikit-learn defaults were left unchanged). The receiver operating characteristic (ROC) curve was obtained (SPSS V.21.0) for the display of the constructed models, then the area under the ROC curve (AUC) was used to designate the ROC effect. Then, to confirm that testing performance does not benefit from potential overfitting of RF classifiers, a five-fold cross-validation was used to investigate prediction errors associated with our models (Zheng et al., 2020).

### Statistical Analysis

Statistical analyses were carried out using SPSS version 21.0 (SPSS, Chicago, IL, United States). For continuous variables such as age, we used the *t*-test or analyzed data by ANOVA followed by LSD’s multiple comparisons tests. We applied non-parametric factorial Wilcoxon rank-sum test or Kruskal–Wallis test followed by Dunn’s multiple comparisons tests to compare two or three groups in case of heteroscedasticity or non-normally distributed variables. Furthermore, categorical data such as sex were analyzed by Chi-square test. Statistical significance level was set at *p* < 0.05.

## Results

### OMG Subjects Exhibited Different Gut Microbiota Versus GMG Subjects

Totally, 31 OMG, 39 GMG subjects and 74 HCs were recruited from our previous study (Zheng et al., 2019a). The detailed clinical characteristics of these subjects were presented in Table 1. We found that the QMG scores were significantly lower (indicating more mild clinical status) in the OMG group than in the GMG group (*P* = 0.002). Besides, there were no significant differences of age and gender among the three groups. Furthermore, these clinical characteristics including BMI, duration of disease, history of thymic hyperplasia were not significantly different between OMG and GMG subjects.

**FIGURE 1:**
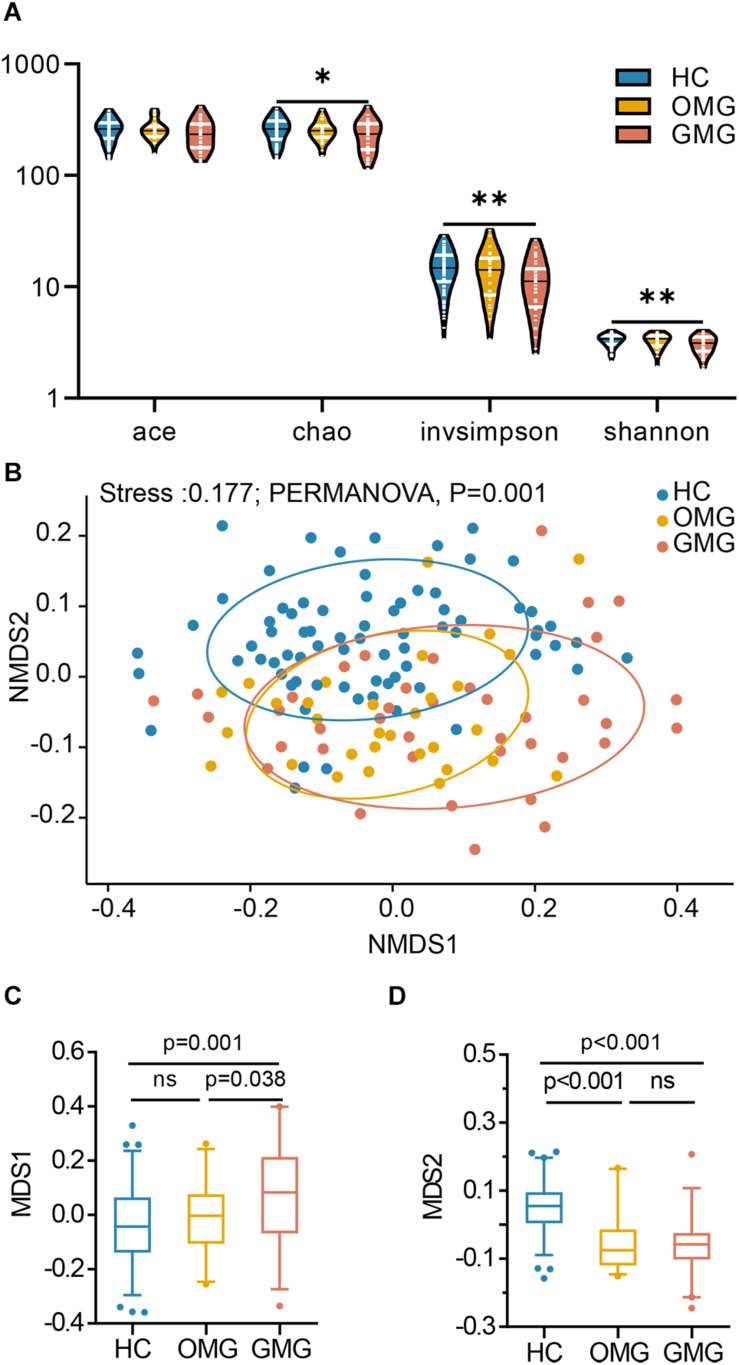
Differential gut microbial characteristics among the GMG, OMG, and HC groups. **(A)** α-phylogenetic analysis revealed that the generalized myasthenia gravis group (GMG, *n* = 39), but not the ocular myasthenia gravis group (OMG, *n* = 31), was characterized by lower bacterial richness (chao, *p* = 0.011 by one way ANOVA) and diversity (invsimpson, *p* = 0.003 by one way ANOVA and Shannon, *p* = 0.007 by the Kruskal–Wallis test) than the healthy control group (HC, *n* = 74). **(B)** NMDS analysis displayed a striking segregation among OMG, GMG and HCs at OUT level (Stress, 0.177; PERMANOVA, *p* = 0.001). **(C)** In the NMDS1, GMG subjects were statistically distinguished from OMG subjects and HCs. **(D)** In the NMDS2, both OMG and GMG were statistically distinguished from HCs (multiple comparisons, one-way ANOVA). Abbreviation: NMDS, non-metric multidimensional scaling.

Here, the alpha diversity indices including microbial community richness (Chao, Ace) and diversity (Shannon, Invsimpson) were compared among the three groups. Consequently, we found that the indices of Ace, Invsimpson and Shannon were depleted in patients with GMG versus HCs. These indices were not different between OMG and GMG cohorts, or the OMG and HC cohorts (Figure 1A). To further investigate whether the overall microbial phenotypes of patients with OMG or GMG were different from that in HCs, beta diversity using the unweighted UniFrac distances was performed. Non-metric multidimensional scaling (NMDS) analysis showed that a striking segregation among OMG, GMG and HCs was displayed at the operational taxonomic units (OTU) level (Stress,0.177; PERMANOVA, *p* = 0.001) (Figure 1B). In the NMDS1, the GMG group was significantly different from OMG and HC groups (*p* = 0.038, GMG versus OMG; *p* = 0.001, GMG versus HCs; one-way ANOVA) (Figure 1C). In addition, both OMG and GMG were significantly different from HC in the NMDS2 (*p* < 0.001, both; one-way ANOVA) (Figure 1D). Otherwise, control analyses showed that the OMG, GMG or HC subjects were not clustered based on gender (Supplementary Figures S1A–C), medication/treatment history (Supplementary Figures S1D,E).

**FIGURE 2:**
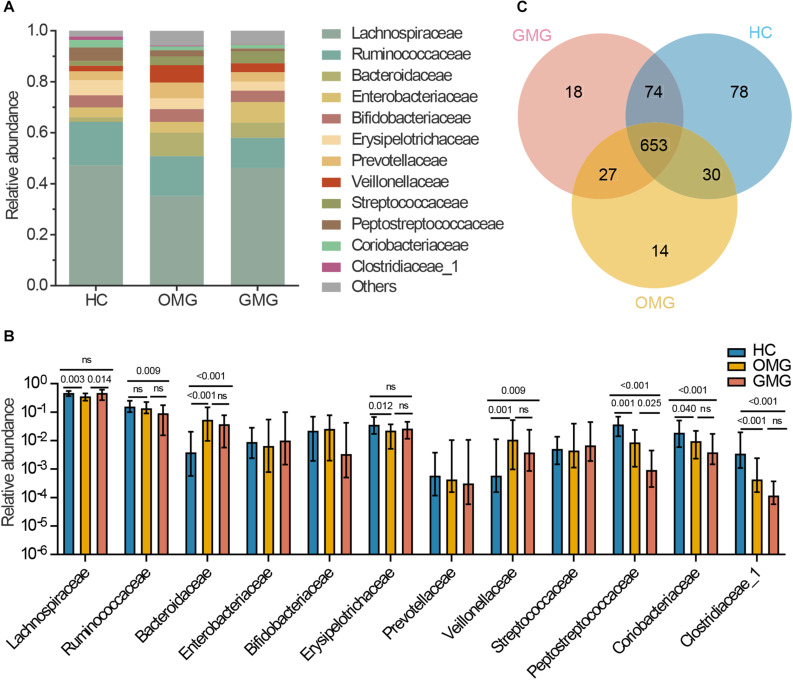
Comparison of the gut microbial composition among the three groups at family and OTU levels. **(A)** The community bar plot illustrated that the gut microbiome was mainly composed of 12 families. **(B)** Families Lachnospiraceae and Erysipelotrichaceae were significantly decreased in OMG group versus HCs, and the relative abundance of Lachnospiraceae in OMG group was also lower than that in GMG group. In addition, Ruminococcaceae was significantly depleted in GMG group versus HCs. Compared to HC group, Peptostreptococcaceae, Coriobacteriaceae and Clostridiaceae_1 were depleted, while Bacteroidaceae and Veillonellaceae were enriched in both OMG and GMG subjects. Moreover, the relative abundance of Peptostreptococcaceae in GMG was lower than that in OMG group. **(C)** A venn diagram demonstrated that 653 of 894 OTUs were discovered among the three groups, whereas 14, 18 and 78 OTUs were specific to OMG (yellow circle), GMG (red circle) and HCs (blue circle), respectively. (Each value represents median with interquartile range, *p*-values were determined by the Kruskal–Wallis test).

Next, to identify the differential gut microbes related to the two subtypes of MG, the relative abundances of microbial compositions were compared among the three groups at family levels (Figure 2). Here, we found that the gut microbiome was mainly composed of 12 families among the three groups (Figure 2A). Among them, the relative abundances of Lachnospiraceae and Erysipelotrichaceae families were lower in OMG than HCs. Furthermore, Lachnospiraceae was also significantly decreased in OMG relative to GMG. For GMG, Ruminococcaceae was significant different (lower) from HCs. In addition, compared to HC group, Peptostreptococcaceae, Coriobacteriaceae and Clostridiaceae_1 were depleted, while Bacteroidaceae and Veillonellaceae were enriched in both OMG and GMG groups. Moreover, family Peptostreptococcaceae was much lower in GMG group than that in OMG group. Further, there were no difference in Bacteroidaceae, Veillonellaceae, Coriobacteriaceae and Clostridiaceae_1 between OMG and GMG groups (Figure 2B).

**FIGURE 3:**
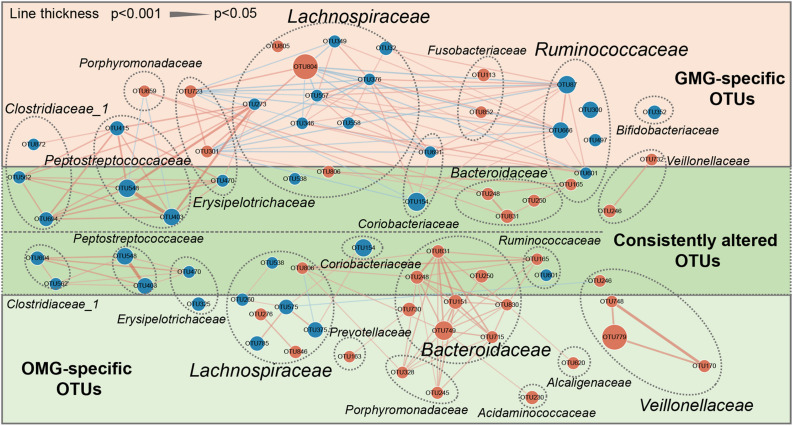
A co-occurrence network inferred from the relative abundances of differential OTUs associated with OMG or GMG. The discriminative OTUs related to OMG or GMG were identified based on LDA ≥ 2.5 and fold change > 2.0. Totally, 71 discriminative OTUs were identified between OMG or GMG and HCs. Among them, 14 of 71 OTUs were identically changed in both OMG and GMG groups versus HC group (dark green area), whereas most of OTUs were specific to OMG (20/34) (light green area) or GMG alone (23/37) (pink area). Compared with HC, OMG-specific OTUs were mainly assigned to the families Lachnospiraceae (6 OTUs), Bacteroidaceae (5 OTUs) and Veillonellaceae (3 OTUs), while GMG-specific OTUs were mainly assigned to the families Lachnospiraceae (9 OTUs) and Ruminococcaceae (4 OTUs). Size of the dots indicates the relative abundance of the OTUs. Red dots represent enriched OTUs in OMG or GMG group relative to HC group; blue dots represent depleted OTUs in OMG or GMG group relative to HC group. OTUs annotated to family level were profiled. Edges between dots represent Spearman’s correlation <- 0.45 (light blue), or >0.45 (light red), edges thickness indicate *p*-value (*p* < 0.05).

To outline the shared and distinct microbial characteristics between OMG and GMG patients at length, we further identified key differential OTUs in OMG or GMG group versus HCs via LEfSe analysis. Overall, we found that 653 of 894 OTUs were discovered in the three groups, while 14, 18 and 78 OTUs were specific to OMG, GMG and HCs, respectively (Figure 2C). Totally, we identified 34 and 37 differential OTUs responsible for distinguishing the OMG versus HCs, and GMG versus HCs, respectively (Supplementary Table S1 and Supplementary Figures S2A,B). Compared to HC group, fourteen OTUs were identically changed in both OMG and GMG groups, which included enriched OTUs belonging to family Bacteroidaceae (3 OTUs) and depleted OTUs belonging to families Clostridiaceae_1 (2 OTUs) or Peptostreptococcaceae (2 OTUs). Additionally, majority of differential OTUs were peculiar to either OMG (20/34) or GMG (23/37) group. Compared with HC, OMG-specific OTUs were mainly assigned to the families Lachnospiraceae (4 decreased and 2 increased OTUs), Bacteroidaceae (5 increased OTUs) and Veillonellaceae (3 increased OTUs), while GMG-specific OTUs were mainly assigned to the families Lachnospiraceae (7 depleted and 2 enriched OTUs) and Ruminococcaceae (4 depleted OTUs) (Figure 3).

In addition, co-occurrence network analysis provided an explanation of the interacting correlation among these changed OTUs (Figure 3). In OMG group, majority of differential OTUs were positively correlated with each other. In GMG group, however, the covariant networks composed of altered OTUs were rather complex and diverse. For instance, we found that an intricate covariant networks were displayed among families Lachnospiraceae, Ruminococcaceae, and Erysipelotrichaceae. Furthermore, all the Peptostreptococcaceae OTUs were positively covaried with all the Clostridiaceae_1 OTUs and one depleted Lachnospiraceae OTU (OTU273).

To further determine the microbial differences between the OMG and GMG subjects, direct distinction between the two groups was carried out. Consequently, most of differential OTUs (9/10) were increased in OMG relative to GMG subjects (Supplementary Table S2 and Supplementary Figure S2C). These increased OTUs mainly belonged to Bacteroidaceae (OTU151 and OTU749), Erysipelotrichaceae (OTU301 and OTU470) and Lachnospiraceae (OTU349 and OTU47) (Supplementary Figure S3). Together, these results further confirmed the difference of microbial composition between GMG and OMG groups.

### The Fecal Metabolome Differed Between Ocular and Generalized MG Patients

**FIGURE 4:**
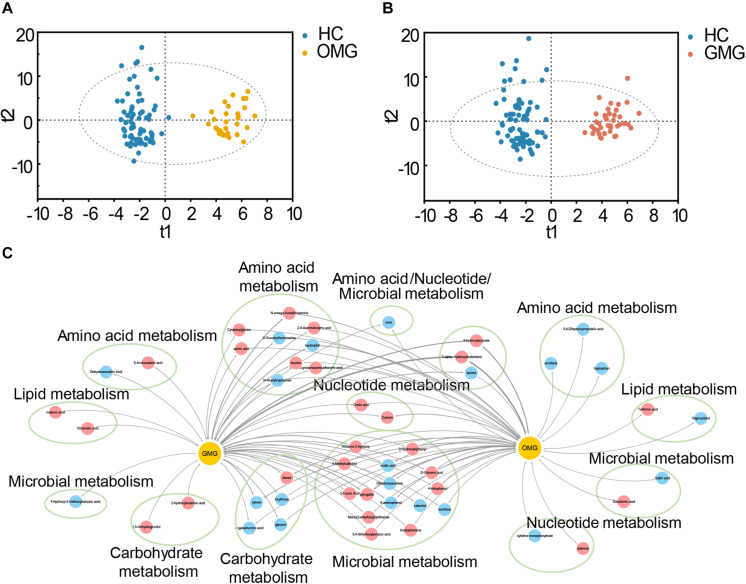
Metabolic characteristics of two subtypes of MG. **(A,B)** The orthogonal partial least-squares discriminant analysis (OPLS-DA) scores plots exhibited a clear separation between the OMG (orange dots, **A**) or GMG (red dots, **B**) subjects and HCs (blue dots). **(C)** The shared and distinct fecal metabolites detected in OMG and GMG subjects versus HCs. These differential metabolites mainly belonged to microbial metabolism, amino acid metabolism, carbohydrate metabolism, lipid metabolism and nucleotide metabolism. Red nodes indicate upregulated metabolites, while blue nodes indicate downregulated metabolites in MG subjects related to HCs. The thickness represents *p*-value (*p* < 0.05).

Metabolomic studies have shown that molecules derived from the microbiota may influence metabolic and behavioral phenotypes in humans (Blumberg and Powrie, 2012; Nicholson et al., 2012). Given that each type of MG individuals displayed different intestinal microbial disorders, we hypothesized that the fecal metabolome would be different between OMG and GMG subjects. Here, we further applied non-targeted metabolomics to characterize the metabolic differences and similarities in OMG and GMG patients related to HCs. The orthogonal partial least-squares discriminant analysis (OPLS-DA) showed that the metabolic signatures of both OMG and GMG groups were substantially different from that in HC group (Figures 4A,B). By analyzing the OPLS-DA loading coefficient plot, a total of 86 differential metabolites were obtained in the two groups (VIP > 1.0, *p* < 0.05 and FDR < 0.1). Intriguingly, majority of these fecal differential metabolites were shared between the two subtypes of MG. A few of differential fecal metabolites were specific to OMG (9/44) or GMG group (7/42), respectively (Supplementary Table S3). Further, functional clustering analysis demonstrated that the majority of these shared metabolites between two subtypes of MG mainly belonged to microbial metabolism, amino acid metabolism and carbohydrate metabolism (Figure 4C). In regard of OMG specific metabolites, three down-regulated metabolites (ornithine, tryptophan and 3,4-Dihydroxymandelic acid) were involved in amino acid metabolism; and one down-regulated (stigmasterol) and one up-regulated (tartronic acid) metabolite belonged to lipid metabolism. In addition, one down-regulated (gallic acid) and one up-regulated (galactonic acid) metabolites were linked with microbial metabolism. Specifically, one decreased (cytidine-monophosphate) and one increased (adenine) metabolites were involved in nucleotide metabolism in OMG cohorts. In contrast, two up-regulated metabolites (malonic acid and citramalic acid) belonged to lipid metabolism, and one down-regulated (dehydroascorbic acid) and one enriched (5-aminovaleric acid) metabolites to amino acid metabolism, and one downregulated metabolite (4-hydroxy-3-methoxybenzoic acid) to microbial metabolism were identified in the patients with GMG relative to HCs. Additionally, two up-regulated metabolites (1,5-anhydroglucitol and 2-hydroxybutanoic acid) belonged to carbohydrate metabolism were also specifically linked with GMG onset.

### Combinatorial Biomarker for Discriminating OMG From GMG Group

**FIGURE 5:**
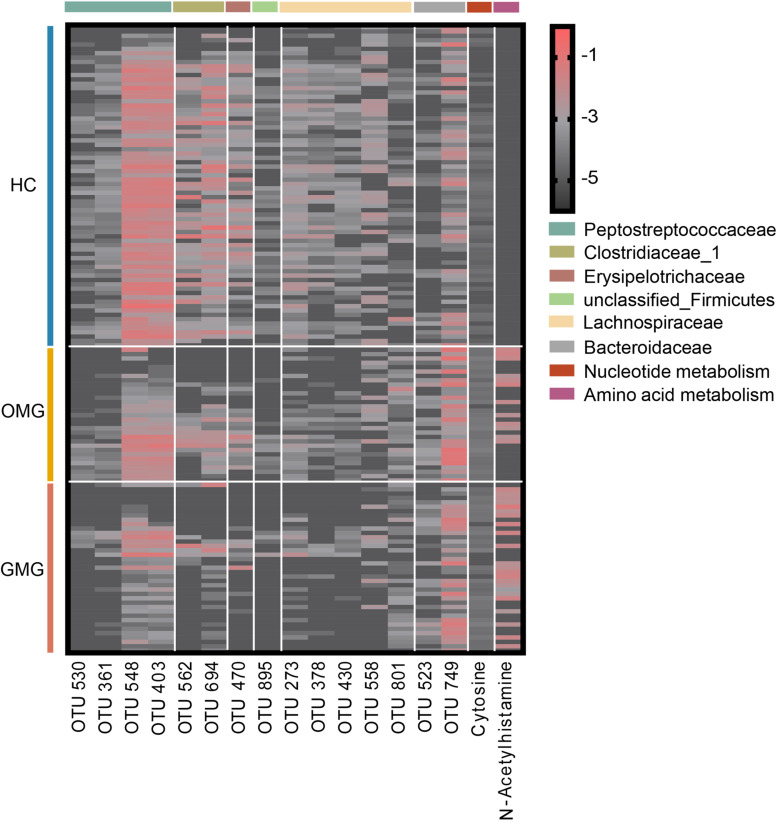
Combinatorial microbiota and metabolite biomarkers for discriminating GMG, OMG, and HC groups. Via linear discriminant analysis (LEfSe), 15 discriminative OTUs responsible for discrimination among the three groups were identified based on LDA score > 2.5. These discriminative OTUs mainly belonged to the families Lachnospiraceae (5 OTUs), Peptostreptococcaceae (4 OTUs), Clostridiaceae_1 (2 OTUs) and Bacteroidaceae (2 OTUs). Meanwhile, based on VIP > 1.0, *p* < 0.05 and FDR < 0.1, cytosine and n-acetylhistamine were significantly different among OMG, GMG and HC.

To identify microbial and metabolic signatures capable of discriminating OMG and GMG from each other, as well as from HCs, discriminative OTUs and metabolites were identified among the three groups (Supplementary Table S4 and Supplementary Figure S2D). Using LEfSe analysis, a total of 15 differential OTUs responsible for discriminating among the three groups were identified based on LDA score > 2.5. These discriminative OTUs mainly assigned to the families Lachnospiraceae (5 OTUs), Peptostreptococcaceae (4 OTUs), Clostridiaceae_1 (2 OTUs) and Bacteroidaceae (2 OTUs) (Figure 5). Meanwhile, by analyzing the OPLS-DA loading coefficient plot, we found that cytosine and n-acetylhistamine were significantly different among three groups based on VIP > 1.0, *p* < 0.05 and FDR < 0.1.

**FIGURE 6:**
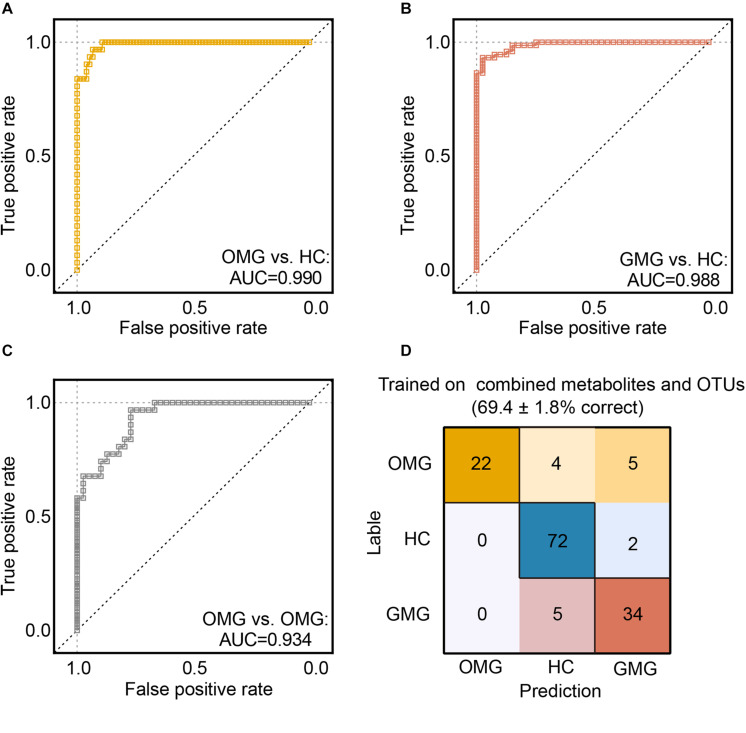
Predicting MG subtypes based on gut microbial and metabolic markers. **(A–C)** We trained random forest (RF) classifiers on discriminative fecal metabolites and microbial OTUs to identify MG subtypes. Receiver operating characteristic (ROC) curves showed that this combinatorial biomarker panel including 15 discriminative OTUs and 2 discriminative fecal metabolites enabled discriminating OMG and GMG from each other, as well as from HCs, with high diagnostic accuracy (OMG vs HC, AUC = 0.990; GMG vs HC, AUC = 0.988; OMG vs GMG, AUC = 0.934). **(D)** “Confusion matrix” evaluations of MG subtype RF classifiers. The number in row i and column j indicated how many samples were labeled as subtype i but assigned to subtype j. A perfect subtype RF classifier (100% accuracy) would have 0 counts for all non-diagonal entries (that is, no misclassified samples). Matrix cells were shaded within-row in proportion to their value (yellow, OMG; red, GMG; blue, HCs). Accuracy values indicated the fraction of correctly classified instances; error values reflect the s.e.m of a proportion. Consequently, the plot also showed that this combinatorial biomarker panel was capable of predicting OMG, GMG or HCs correctly 69.4 ± 1.8% of the time.

Next, we trained random forest (RF) classifiers on above discriminative metabolites and OTUs to discriminate MG subtypes. Receiver operating characteristic (ROC) curves were used to quantify their diagnostic performance. Consequently, we found that this combinatorial biomarker panel enabled discriminating OMG and GMG from each other, as well as from HCs, with high diagnostic accuracy (OMG versus HC, AUC = 0.990; GMG versus HC, AUC = 0.988; OMG versus GMG, AUC = 0.934) (Figures 6A–C). In addition, we also observed that this biomarker panel could predict OMG, GMG or HC labels correctly 69.7 ± 1.8% of the time. The most common source of annotation error was classifying patients with OMG or GMG as HC, or classifying OMG as GMG. Comparatively, patients with GMG and healthy control individuals were rarely classified into OMG patients, while healthy control individuals were sometimes erroneously classified as patients with GMG (Figure 6D).

## Discussion

In this study, we compared the microbial and metabolic characteristics between OMG and GMG. And we firstly outlined the shared and distinct microbial and metabolic signatures between two groups. Here, we found that the microbial composition of OMG was substantially different from that of patients with GMG. For example, our findings showed that patients with GMG displayed lower α-phylogenetic diversity compared with HCs. Indeed, a greater proportion of OMG subjects presented with higher community richness and diversity compared with the GMG cohort, although the quantified levels detected were intermediate to those observed in GMG and HC groups. Generally speaking, high α-diversity is equal to a “good” health status (Huttenhower et al., 2012). Therefore, our findings suggested that gut microbial disturbances in patients with GMG were more severe than that in patients with OMG, which was consistent with clinical presentation of two subtypes of MG.

Further, compared with HC individuals, families Bacteroidaceae and Veillonellaceae enriched in both OMG and GMG subjects, while families Lachnospiraceae and Ruminococcaceae depleted in OMG and GMG, respectively. Consistent with our findings, previous studies have demonstrated that Enterobacteriaceae, Bacteroidales and Veillonellaceae positively correlated with some autoimmune diseases, whereas Lachnospiraceae and Ruminococcaceae, typically producing short chain fatty acids (SCFA) (Yilmaz et al., 2019), negatively correlated with these diseases (Gevers et al., 2014; Clemente et al., 2018). These findings suggested that disturbances of gut microbes may synergistically modulate the development of OMG and GMG.

We also revealed the shared and distinct microbial characteristics between OMG and GMG at the OTU level. Meanwhile, complex covariant networks were presented among these discriminative OTUs, which may provide mechanistic insights. For instance, upregulated Bacteroidaceae OTUs and other OTUs that were positively correlated with them, constructed an idiosyncratic covariant network mostly from the OMG-specific OTUs. Moreover, three upregulated Veillonellaceae OTUs positively covaried with each other, which also played a cooperative role in the gut microbial environment of OMG. In GMG group, however, the correlation networks constructed by altered OTUs were rather complex and diverse. Among them, an intricate covariant network was generated with major downregulated Lachnospiraceae OTUs and all the depleted Ruminococcaceae OTUs. Importantly, previous studies have explored that Enterobacteriaceae, Bacteroidales and Veillonellaceae were thought to contribute to perturbations in the immune function, but Lachnospiraceae and Ruminococcaceae were associated with lower levels of inflammation (Gevers et al., 2014; Clemente et al., 2018). Together, we deduced that microbial composition of patients with OMG was characterized by enriched families Bacteroidaceae and Veillonellaceae, yet the patients with GMG were mainly linked with decreased family Lachnospiraceae.

Overall, we found that both OMG and GMG were mainly involved in disturbances of nucleotide metabolism, amino acid metabolism, carbohydrate metabolism and microbial metabolism. The disturbances of fecal metabolism partly confirmed the altered microbial composition in the OMG and GMG groups. Previously, it was well-known that the MG is an autoimmune disease. Here, our findings suggest that the influence of altered microbial composition on fecal metabolisms may be linked with OMG and GMG. However, it should be admitted that how these alterations in intestinal metabolism attribute to onset of OMG and GMG remains unknown. We speculate that these following possibilities are worth to explore: (i) previous studies showed that patients with MG were associated with alternations of antioxidant markers (Fuhua et al., 2012; Yang et al., 2016). It is widely accepted that nucleotide metabolism can modulate the oxidative stress. Thus, it is likely that microbial nucleotide metabolism may participate in the development of OMG and GMG via modulation of host’s oxidative stress; (ii) Recently, Blackmore et al. (2020) found that MG was associated with disturbances of serum phenylalanine and tyrosine metabolisms. Here, we found that both OMG and GMG individuals were linked with altered fecal amino acid metabolism. As some of metabolites have immune-metabolomic properties, further studies to integrate the fecal and serum metabolic changes is required to uncover the underlying roles of microbial metabolites in the MG onset; (iii) previous studies have shown that gut microbiome played a vital role in energy regulation and metabolism (Nieuwdorp et al., 2014), which may account for the disturbances of carbohydrate metabolism in MG. Interestingly, we found that adenine and cytidine-monophosphate specific to OMG subjects were involved in nucleotide metabolism; 3,4-dihydroxymandelic acid, ornithine, tryptophan 5-aminovaleric acid and dehydroascorbic acid specific to OMG or GMG belonged to amino acid metabolism; 2-hydroxybutanoic acid and 1,5-anhydroglucitol specific to GMG subjects were linked with carbohydrate metabolism. Thus, we deduced that gut microbiota played an important but complex roles in fecal metabolism of OMG and GMG subjects.

Here, we identified a combinatorial biomarker composing of 15 bacterial OTUs and 2 metabolites including Cytosine and N-Acetylhistamine, which could distinguish OMG and GMG individuals with AUC values ranging from 0.934 to 0.990. These discriminative OTUs mainly belonged to the families Lachnospiraceae (OTU273, OTU378, OTU430, OTU558, OTU801), Peptostreptococcaceae (OTU361, OTU403, OTU530, OTU548), Clostridiaceae_1 (OTU562, OTU694) and Bacteroidaceae (OTU523, OTU749). Furthermore, this combinatorial biomarker was able to predict OMG, GMG or HC labels correctly about 69.7% of the time.

This study has the following limitations: (i) due to lack of blood samples, how the gut microbiome shapes the blood signatures remains unknown. Further studies to integrate the fecal and blood metabolic signatures are valuable to deeply understand the similarities and differences of microbial function in the OMG and GMG; (ii) due to limited resolution of 16S rRNA sequencing method, further studies using the shotgun metagenomics should be performed to identify the definitive bacterial species and microbial function linked with OMG and GMG; (iii) the discriminative power of biomarker panel should be validated using samples from multicenter cohorts; (iv) due to the long duration of MG, future studies should explore the alteration in gut microbiota of patients with MG who are newly diagnosed and drug-naïve, to further validate our findings.

In summary, we found that gut microbiota and fecal metabolism were significantly different between patients with OMG and GMG. And we identified gut microbes and metabolites specific to two subtypes of MG. Furthermore, we identified a combinatorial biomarker panel enabled discriminating OMG and GMG (from each other and from HC) with high accuracy. Taken together, our findings provide a new entry-point to understand the similarities and differences of microbial composition and function in OMG and GMG, which is required to be further clarified in animal studies.

## Data Availability Statement

The raw DNA sequence data were deposited in the National Center for NCBI Sequence Read Archive (https://www.ncbi.nlm.nih.gov/bioproject/PRJNA660322).

## Ethics Statement

The studies involving human participants were reviewed and approved by the Ethics Committee of Chongqing Medical University. The patients/participants provided their written informed consent to participate in this study.

## Author Contributions

XT and PX designed the experiments. XT, YH, TC, YL, JW, WL, BY, HZ, KC, and JD conducted the experiments, analyzed and interpreted the data. GY and XZ collected the clinical samples. XT and YH drafted the manuscript. XT, YH, PZ, and PX revised the manuscript. All authors read the manuscript and approved the final version.

## Conflict of Interest

The authors declare that the research was conducted in the absence of any commercial or financial relationships that could be construed as a potential conflict of interest.

## Footnotes

1 http://qiime.org/

2 http://drive5.com/usearch/manual/uchime_algo.html

3 http://drive5.com/uparse/

4 http://huttenhower.sph.harvard.edu/galaxy/

## Supplementary Material

FIGURE S1 Impact of confounding variables on global gut microbial phenotypes. The global microbial phenotypes of two subtypes of MG groups were not clustered based on gender (*n* = 44, HC Female; *n* = 30, HC Male; *n* = 13, OMG Female; *n* = 18, OMG Male; *n* = 26, GMG Female; *n* = 13, GMG Male) **(A–C)** and medication (*n* = 12, OMG IT; *n* = 19, OMG Non-_IT; *n* = 19, GMG IT; *n* = 20, GMG Non-_IT. IT: immunosuppressive treatment) **(D,E)**.

FIGURE S2 Discriminative OTUs observed in the pairwise comparisons among HC, OMG and GMG groups. Using LEfSe analysis, differential OTUs responsible for discrimination among the three groups were identified based on LDA score > 2.5 and fold change > 2. **(A)** 34 OTUs attributed to distinguishing OMG from HCs. **(B)** 37 OTUs were responsible for distinguishing GMG from HCs. **(C)** 10 OTUs accounted for distinguishing GMG from OMG. **(D)** 15 OTUs were differentially expressed among the three groups.

FIGURE S3 A co-occurrence network inferred from the relative abundances of differential OTUs between OMG and GMG. These differential OTUs were identified based on LDA > 2.5 and fold change > 2. Totally, 10 OTUs were responsible for this discrimination. Compared with GMG group, these increased OTUs in OMG group were mainly assigned to families Bacteroidaceae, Erysipelotrichaceae and Lachnospiraceae. Blue dots, increased microbes in OMG; red dots, increased microbes in GMG. OTUs classified to family level were profiled. Edges between dots represent Spearman’s correlation < −0.35 (light blue), or > 0.35 (light red), edges thickness indicate *p*-value (*p* < 0.05).

TABLE S1 The discriminative OTUs between two subtypes of MG patients and HCs.

TABLE S2 The discriminative OTUs between OMG and GMG patients.

TABLE S3 Differential metabolites between the two subtypes of MG patients and HCs.

TABLE S4 The discriminative OTUs and metabolites among the three groups.
