# Supplementary figures and images for: Differential Gut Microbiota and Fecal Metabolites Related With the Clinical Subtypes of Myasthenia Gravis
Source: Front Microbiol. 2020 Sep 8;11:564579. doi: 10.3389/fmicb.2020.564579 (PMC7506099; doi:10.3389/fmicb.2020.564579)

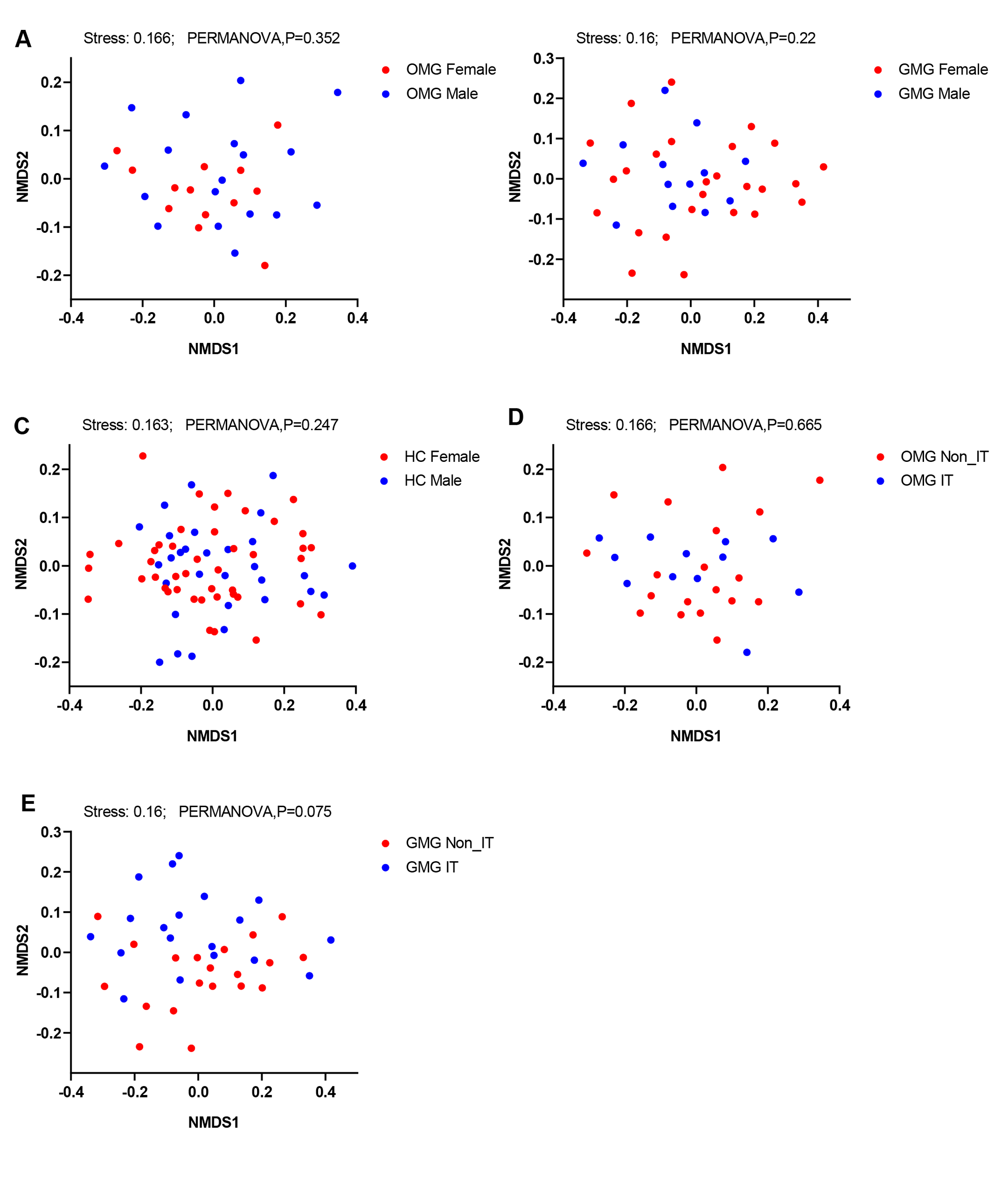

Supplement: FIGURE S1 — Impact of confounding variables on global gut microbial phenotypes. The global microbial phenotypes of two subtypes of MG groups were not clustered based on gender (n = 44, HC Female; n = 30, HC Male; n = 13, OMG Female; n = 18, OMG Male; n = 26, GMG Female; n = 13, GMG Male) (A–C) and medication (n = 12, OMG IT; n = 19, OMG Non-_IT; n = 19, GMG IT; n = 20, GMG Non-_IT. IT: immunosuppressive treatment) (D,E). [file Image_1.TIF]

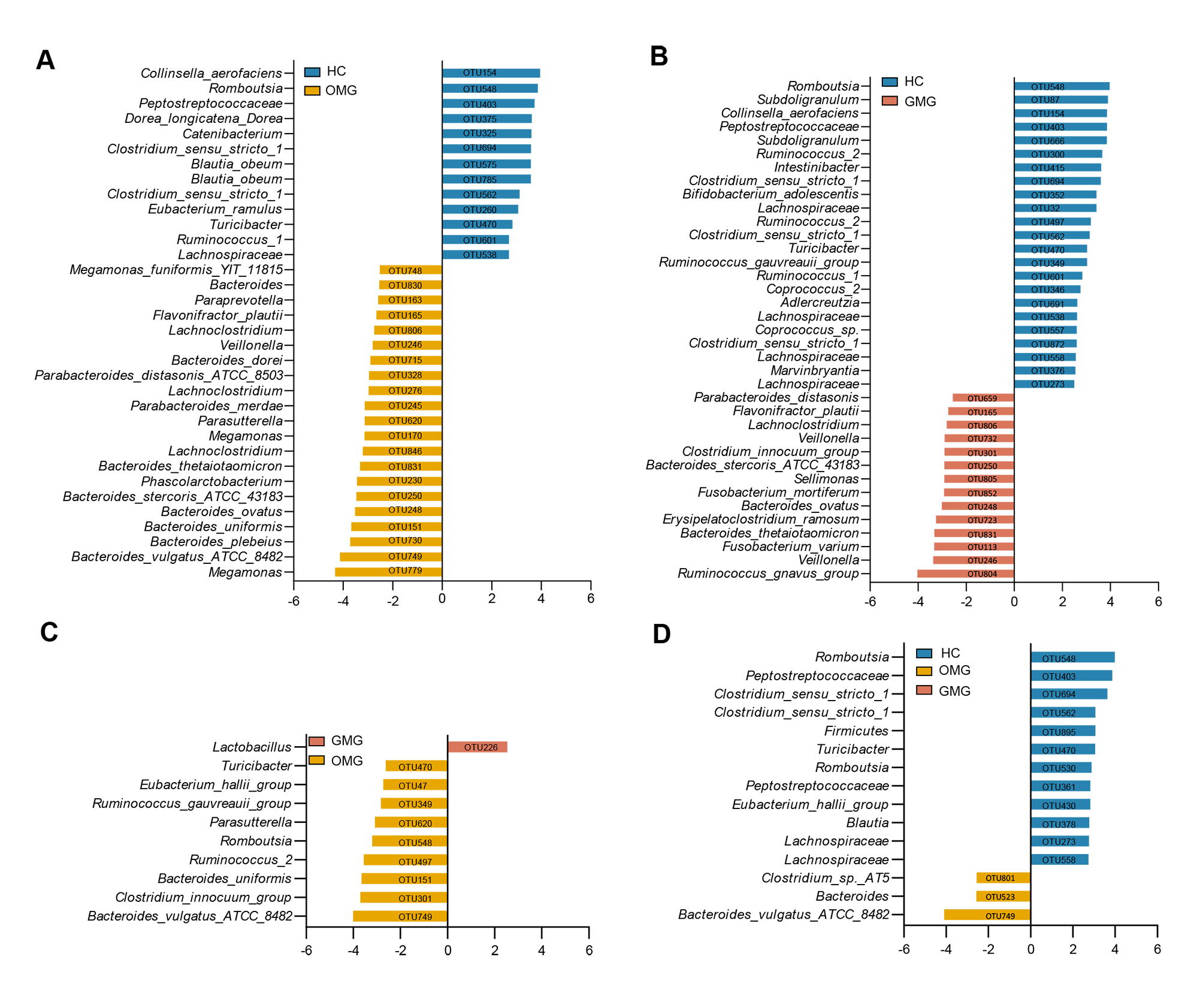

Supplement: FIGURE S2 — Discriminative OTUs observed in the pairwise comparisons among HC, OMG and GMG groups. Using LEfSe analysis, differential OTUs responsible for discrimination among the three groups were identified based on LDA score > 2.5 and fold change > 2. (A) 34 OTUs attributed to distinguishing OMG from HCs. (B) 37 OTUs were responsible for distinguishing GMG from HCs. (C) 10 OTUs accounted for distinguishing GMG from OMG. (D) 15 OTUs were differentially expressed among the three groups. [file Image_2.TIF]

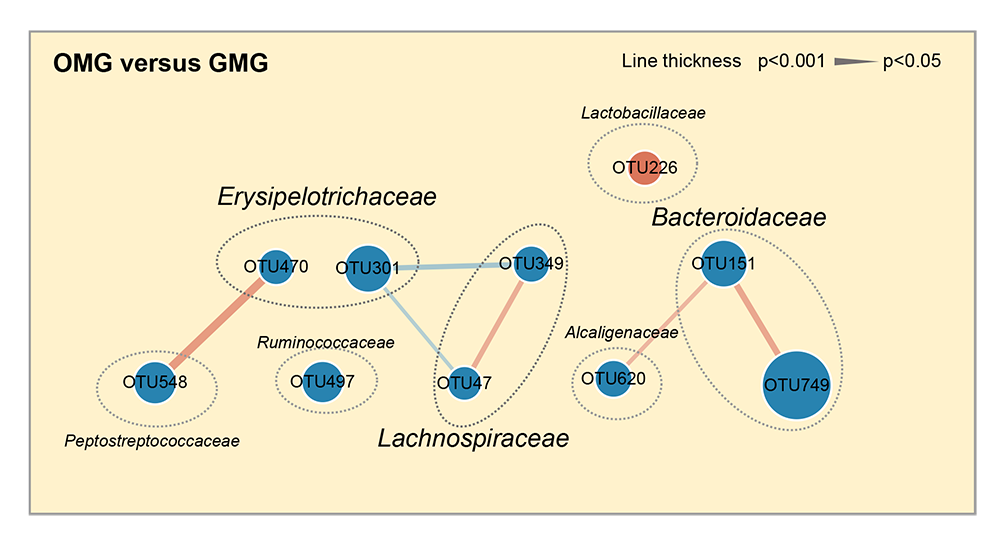

Supplement: FIGURE S3 — A co-occurrence network inferred from the relative abundances of differential OTUs between OMG and GMG. These differential OTUs were identified based on LDA > 2.5 and fold change > 2. Totally, 10 OTUs were responsible for this discrimination. Compared with GMG group, these increased OTUs in OMG group were mainly assigned to families Bacteroidaceae, Erysipelotrichaceae and Lachnospiraceae. Blue dots, increased microbes in OMG; red dots, increased microbes in GMG. OTUs classified to family level were profiled. Edges between dots represent Spearman’s correlation < −0.35 (light blue), or > 0.35 (light red), edges thickness indicate p-value (p < 0.05). [file Image_3.TIF]
