# Supplementary material for: Differential Gut Microbiota and Fecal Metabolites Related With the Clinical Subtypes of Myasthenia Gravis
Source: Front Microbiol. 2020 Sep 8;11:564579. doi: 10.3389/fmicb.2020.564579 (PMC7506099; doi:10.3389/fmicb.2020.564579)
Supplement: Supplementary file 8 [file Data_Sheet_1.pdf]

Supplementary Material

Supplementary Figures

FIGURE S1| Impact of confounding variables on global gut microbial phenotypes. The global microbial phenotypes of two subtypes of MG groups were not clustered based on gender (n=44, HC Female; n=30, HC Male; n=13, OMG Female; n=18, OMG Male; n=26, GMG Female; n=13, GMG Male)(A-C) and medication (n=12, OMG IT; n=19, OMG Non_IT; n=19, GMG IT; n=20, GMG Non_IT. IT: immunosuppressive treatment)(D-E).

FIGURE S2| Discriminative OTUs observed in the pairwise comparisons among HC, OMG and GMG groups. Using LEfSe analysis , differential OTUs responsible for discrimination among the three groups were identified based on LDA score >2.5 and fold change >2. **(A-C)** Totally, 34 ,37 and 10 OTUs attributed to distinguish the OMG versus HCs, GMG versus HCs, and GMG and OMG, respectively. **(D)**15 OTUs were differentially expressed among the three groups.

FIGURE S3| Co-occurrence networks deduced from the relative abundance of differential OTUs between OMG and GMG. The differential OTUs between OMG and GMG groups were identified by LDA (LDA>2.5 and fold change>2). Totally, 10 OTUs were responsible for this discrimination. Compared to GMG group, the OMG group was mainly characterized by altered covarying OTUs belonging to Bacteroidaceae , Erysipelotrichaceae and Lachnospiraceae. Blue dots, increased microbes in OMG ;red dots, increased microbes in GMG. OTUs annotated to family level were marked. Edges between nodes indicate Spearman’s correlation < - 0.35 (light blue), or >0.35 (light red), edges thickness indicate p value (p<0.05).

Supplementary Tables

TABLE S1| The discriminative OTUs between two subtypes of MG patients and HCs.

TABLE S2| The discriminative OTUs between OMG and GMG patients.

TABLE S3| Differential metabolites between the two subtypes of MG patients and HCs.

TABLE S4| The discriminative OTUs and metabolites among the three groups.
